# Production of medium-chain fatty acids and higher alcohols by a synthetic co-culture grown on carbon monoxide or syngas

**DOI:** 10.1186/s13068-016-0495-0

**Published:** 2016-04-02

**Authors:** Martijn Diender, Alfons J. M. Stams, Diana Z. Sousa

**Affiliations:** Laboratory of Microbiology, Wageningen University, Wageningen, The Netherlands; Centre of Biological Engineering, University of Minho, Braga, Portugal

**Keywords:** Butyrate, Caproate, Hexanol, Butanol, *Clostridium kluyveri*, *Clostridium autoethanogenum*, Hydrogen

## Abstract

**Background:**

Synthesis gas, a mixture of CO, H_2_, and CO_2_, is a promising renewable feedstock for bio-based production of organic chemicals. Production of medium-chain fatty acids can be performed via chain elongation, utilizing acetate and ethanol as main substrates. Acetate and ethanol are main products of syngas fermentation by acetogens. Therefore, syngas can be indirectly used as a substrate for the chain elongation process.

**Results:**

Here, we report the establishment of a synthetic co-culture consisting of *Clostridium autoethanogenum* and *Clostridium kluyveri*. Together, these bacteria are capable of converting CO and syngas to a mixture of C_4_ and C_6_ fatty acids and their respective alcohols. The co-culture is able to grow using solely CO or syngas as a substrate, and presence of acetate significantly stimulated production rates. The co-culture produced butyrate and caproate at a rate of 8.5 ± 1.1 and 2.5 ± 0.63 mmol/l/day, respectively. Butanol and hexanol were produced at a rate of 3.5 ± 0.69 and 2.0 ± 0.46 mmol/l/day, respectively. The pH was found to be a major factor during cultivation, influencing the growth performance of the separate strains and caproate toxicity.

**Conclusion:**

This co-culture poses an alternative way to produce medium-chain fatty acids and higher alcohols from carbon monoxide or syngas and the process can be regarded as an integration of syngas fermentation and chain elongation in one growth vessel.

## Background

Over the last decade, synthesis gas (syngas) fermentation has gained attention because of its potential to convert a large variety of waste materials to bio-based chemicals [1]. Additionally, it is possible to convert pure CO_2_ and water into syngas via high temperature co-electrolysis, which can be supplied with electricity and heat derived solely from solar power [2].

Syngas fermentation to acetate and ethanol is relatively well studied, and the array of possible products is rapidly expanding [3]. Bio-based production of medium-chain fatty acids (MCFA), such as butyrate and caproate, is of potential interest because they can serve as commodity chemicals. Additionally, their respective alcohols—butanol and hexanol—could serve as potential biofuels. Butyrate has been shown to be produced naturally from CO by *Eubacterium limosum* [4] and *Butyribacterium methylotrophicum* [5]. Additionally, a pure culture of *Clostridium carboxidivorans* formed butyrate and caproate from CO after medium optimization [6]. Production of higher alcohols from syngas has been reported for genetically engineered clostridia [7, 8], mixed cultures fed with butyrate, caproate, and syngas [9, 10], and several pure cultures of carboxydotrophic bacteria [11–13]. Genetic engineering is one of the approaches to enhance strain production capabilities because most of the wild-type strains have low production rates and yields. For clostridia, the most anticipated syngas biocatalysts, genetic systems are being quickly developed [14]. However, despite recent developments, options to perform metabolic engineering in carboxydrotrophs are still rather limited.

Here, we report the use of a synthetic co-culture of *Clostridium autoethanogenum* (DSM 10061) and *Clostridium kluyveri* (DSM 555) to convert CO or syngas into MCFA and their respective alcohols. *C. autoethanogenum* is one of the model organisms for syngas metabolism and is known for its excellent properties to convert CO or syngas to ethanol and acetate (Table 1) [15]. *C. kluyveri* is found in ruminal environments [16], and is reported to stimulate the production of MCFA in the rumen [17]. It also represents a major fraction of microorganisms in systems performing chain elongation [18]. *C. kluyveri* is well known for its reversed β-oxidation metabolism, converting short chain fatty acids with ethanol into MCFA and hydrogen (Table 1). We hypothesize that a co-culture approach might become an upcoming route to produce MCFA from syngas. Besides, it could also serve as a model and provide insight on how the carboxylate platform, operated with mixed cultures, performs using syngas as electron donor.

**Table 1:** Summary of reactions performed by *C. autoethanogenum* and *C. kluyveri*

	Product	Reaction
*Clostridium autoethanogenum*	Acetate	4 CO + 2 H_2_O ⟶ CH_3_COO^−^ + H^+^ + 2 CO_2_
	Ethanol	6 CO + 3 H_2_O ⟶ C_2_H_5_OH + 4 CO_2_
	Alcohols indirect^a^	2 CO + H_2_O + X_n_–COOH + H^+^ ⟶ X_n_–CH_2_OH + 2 CO_2_
*Clostridium kluyveri*	Butyrate^b^	6 C_2_H_5_OH + 4 CH_3_COO^−^ ⟶ 5 C_3_H_7_COO^−^ + H^+^ + 3 H_2_O + 2 H_2_
	Caproate^b^	6 C_2_H_5_OH + 5 C_3_H_7_COO^−^ ⟶ 5 C_5_H_11_COO^−^ + CH_3_COO^−^ + H^+^ + 3 H_2_O + 2 H_2_

^a^ *X*_n_ displays a saturated carbon chain of length *n*

^b^ Reaction stoichiometry of butyrate and caproate formation might differ based on the concentrations of substrates available

## Methods

### Microorganisms and cultivation

*Clostridium autoethanogenum* (DSM 10061) and *Clostridium kluyveri* (DSM 555) were purchased from the DSMZ strain collection (Braunschweig, Germany). *C. autoethanogenum* and *C. kluyveri* were initially cultivated in DSM-640 and DSM-52 medium, respectively. For co-cultivation, a new medium was designed containing (per liter of medium): 0.9 g NH_4_CL, 0.9 g NaCl, 0.2 g MgSO_4_·7H_2_O, 0.75 g KH_2_PO_4_, 1.94 g K_2_HPO_4_·3H_2_O, 0.02 g CaCl_2_, and 0.5 mg resazurin. The medium was supplemented with the following trace elements (per liter of medium): 1.5 mg FeCl_2_·4 H_2_O, 0.025 mg FeCl_3_·6 H_2_O, 0.070 mg ZnCl_2_, 0.1 mg MnCl·4 H_2_O, 0.006 mg H_3_BO_3_, 0.190 mg CoCl_2_·6H_2_O, 0.002 mg CuCl_2_·2 H_2_O, 0.024 mg NiCl_2_·6 H_2_O and 0.056 mg Na_2_MoO_4_·2 H_2_O, 0.0035 mg, Na_2_SeO_3_, and 0.2 mg Na_2_WO_4_. The medium was boiled and cooled on ice under N_2_ flow, after which 0.75 g l-cysteine was added per liter of medium as reducing agent. Unless stated otherwise, the pH was set to six using NaOH and HCl. Reduced medium was dispensed, under continuous N_2_ flow, into bottles that were immediately capped with rubber stoppers and aluminum caps. The headspace was filled with the desired gas (e.g., CO, H_2_/CO_2_) to a final pressure ranging from 100 to 150 kPa, depending on the experiment. Bottles were autoclaved immediately after preparation. Before inoculation, the medium was further supplemented with a vitamin solution in a 1:50 dilution, containing per liter: 1 mg biotin, 10 mg nicotinamide, 5 mg *p*-aminobenzoic acid, 10 mg thiamin, 5 mg pantothenic acid, 25 mg pyridoxamine, 5 mg cyanocobalamine, and 5 mg riboflavine. Yeast extract, trypticase peptone, ethanol, and acetate were added from sterile stock solutions. Initial incubations for co-cultivation were done at a concentration of 1 g/l yeast extract and 1 g/l peptone. Subsequent transfers and characterization experiments were performed in presence of 0.5 g/l yeast extract and in absence of peptone. Unless stated otherwise, cultivation was done non-shaking at 37 °C. Unless stated otherwise, pure cultures were incubated as follows: *C. kluyveri* was grown with 90 mM ethanol and 80 mM acetate in presence of 10 kPa CO_2_, and *C. autoethanogenum* was grown with 130 kPa CO as sole substrate.

### Co-culture experiments

Initial co-culture experiments were performed in 250 ml bottles with 70 ml liquid phase. *C. autoethanogenum* and *C. kluyveri* were transferred from actively growing cultures in exponential phase to the designed medium. Pre-cultures of *C. autoethanogenum* were incubated at 150 rpm shaking in presence of 80 mM acetate under a headspace of 100 kPa CO and 50 kPa H_2_. Pre-cultures of *C. kluyveri* were grown non-shaking in absence of CO. After detection of growth in both pure cultures, 35 ml of each culture was inoculated into the other culture, initiating the co-cultivation. Immediately, after initiation of co-cultivation, the headspace of the CO and H_2_ containing bottles was re-pressurized with CO and H_2_. In bottles initially containing no CO or H_2_, 50 kPa CO was added. The bottles were further incubated non-shaking at 37 °C. After detection of growth of both organisms in the co-cultures via liquid and gas profile analysis and microscopic observation, 0.5 ml of the co-cultures was transferred to new 250 ml bottles containing 70 ml medium with 80 mM acetate and 130 kPa CO. The co-culture was further maintained under these conditions, requiring transfer every 14 days.

All characterization tests were performed using 120 ml bottles containing 35 ml liquid. For tests requiring acetate, butyrate, or caproate, stock solutions were used which were made anaerobic via N_2_ flushing and set at pH 6 using NaOH and HCl. In case of re-addition of CO during the experiment, four cycles of flushing with pure CO were applied, using a 0.22-µm filter to keep the gas flow sterile. When assessing the effect of shaking conditions, 150 rpm shaking was applied in all cases. For characterizing the production profile in presence of excessive amounts of CO, bottles with 1140 ml total volume were used, containing 100 ml medium and a 110 kPa CO headspace. Culture inoculation was done in 1:100 ratio with an actively growing co-culture. The bottles were initially incubated non-shaking and shaking was applied after ethanol-limited butyrate production became apparent. Product and substrate profiles were in all cases assessed using HPLC and GC.

### Analytical techniques

Liquid phase composition was analyzed via high pressure liquid chromatography equipped with a MetaCarb 67H column (Agilent Technologies, Santa Clara, CA). The column was operated at a temperature of 45 °C at a flow rate of 0.8 ml/min. Detection was done via a RI and UV detector. 0.01N H_2_SO_4_ was used as eluent. In all cases, samples of 0.5 ml were taken and immediately centrifuged at 13,000*g*. Subsequently 0.4 ml supernatant was added to 0.6 ml 10 mM DMSO in 0.1N H_2_SO_4_. Concentrations below 0.3 mM could not accurately be quantified and are further referred to as trace amounts.

For gas analysis, gas samples of 0.2 ml were taken with a 1-ml syringe and analyzed in a Compact GC 4.0 (Global Analyser Solutions, The Netherlands). CO and H_2_ were measured using a molsieve 5A column operated at 100 °C coupled to a Carboxen 1010 pre-column. CO_2_ was measured using a Rt-Q-BOND column operated at 80 °C. Detection was in all cases done via a thermal conductivity detector.

### Model fitting and production rate estimation

Production rates of the co-culture were estimated by non-linear data fitting to a modified Gompertz model (Eq. 1) [19]. To estimate the net production rates, the derivative of the modified Gompertz model was used (Eq. 2), in which *A* represents the maximal concentration of product reached (mM), *V*_m_ indicates the maximal volumetric production rate (mmol/l/day), and γ is a representation of the lag time before production occurs (days). Standard errors of the determined parameters were translated to standard errors of the production rate via error propagation.

1 $$f\left( t \right) = A{\text{e}}^{{ - e^{{\frac{{V_{\text{m}} e}}{A}(\gamma - t) + 1}} }}$$

2 $$f^{'}\left(t \right) = eV_{\text{m}} {\text{e}}^{{ - e^{{\frac{{V_{\text{m}} e}}{A}(\gamma - t) + 1}} }} {\text{e}}^{{\frac{{V_{\text{m}} e}}{A}(\gamma - t) + 1}}$$

## Results

**Fig. 1:**
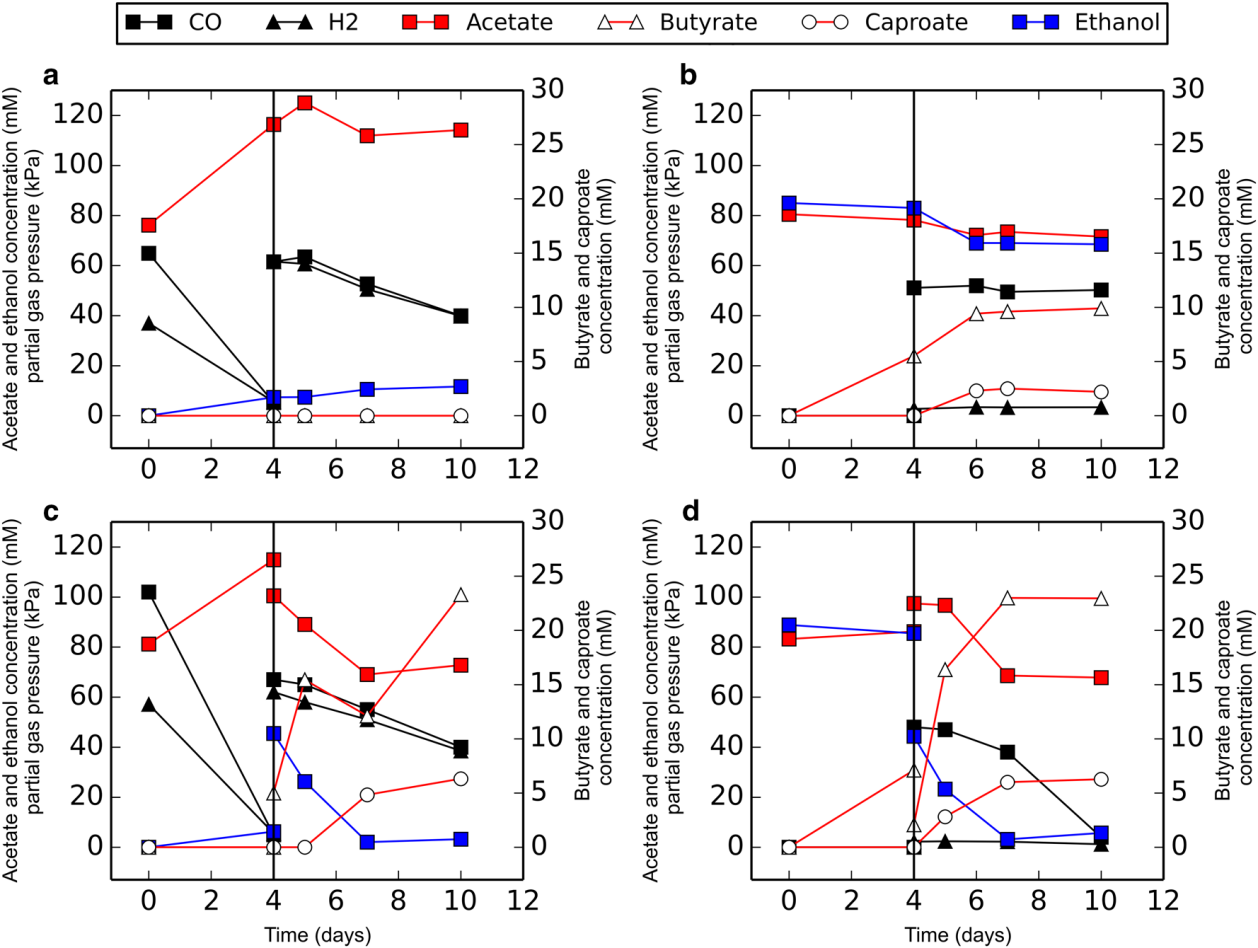
Co-culture establishment. **a** Production profile of *C. autoethanogenum* grown with CO and H_2_, the headspace was refilled with H_2_/CO at *t* = 4. **b** Production profile of *C. kluyveri*, at *t* = 4, 50 kPa CO was introduced to the culture. **c** A pure culture of *C. autoethanogenum* mixed in a 1:1 ratio with a pure culture of *C. kluyveri* at *t* = 4. **d** A pure culture of *C. kluyveri* mixed in a 1:1 ratio with a pure culture of *C. autoethanogenum* at *t* = 4. The legend is representative for all displayed *graphs*. *Solid* and *open* *circle* symbols represent *left* and *right* *y-axis*, respectively

*Clostridium autoethanogenum* and *C. kluyveri* both grew efficiently in the designed medium. *C. autoethanogenum* grown on CO/H_2_ formed acetate and ethanol, and chain-elongated products were not formed (Fig. 1a). Pure cultures of *C. kluyveri* utilized ethanol and acetate as substrate, forming butyrate, caproate, and hydrogen as end products. Introduction of 50 kPa CO in pure cultures of *C. kluyveri* inhibited its activity (Fig. 1b). Some chain-elongated products accumulated, but consumption of acetate and ethanol halted before they were depleted. Upon initiation of co-cultivation by adding both monocultures together in 1:1 ratio, carboxydotrophic and chain elongating activity was observed (Fig. 1c, d). Trace amounts of butanol and hexanol were detected in the co-culture, while these compounds were not observed in any of the monocultures incubated with CO, acetate, and ethanol.

**Fig. 2:**
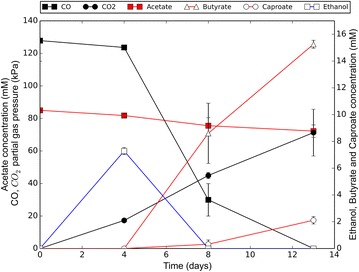
Standard production profile of the co-culture in non-shaking conditions. On all data series, a standard deviation is displayed over duplicate experiments. *Solid* and *open circle* symbols represent *left* and *right y-axis*, respectively

Co-cultures could be maintained and transferred stably by incubating non-shaking under CO or CO/H_2_ headspace in presence of 80 mM acetate (Fig. 2). The co-culture was capable of growing efficiently with 0.5 g/l yeast extract. Lower concentrations of yeast extract had a strong negative effect on the production rates, and significantly increased the lag phase. Studies have shown that it is possible to grow both *Clostridium* strains in absence of yeast extract after an adaptation period [20, 21]. However, as the main focus of this study was on establishing co-cultivation, it was chosen to keep the yeast extract at 0.5 g/l to ensure non-stringent growth conditions for both organisms. A pH range from 7 to 4 was tested to assess the co-culture tolerance, yielding a functional co-culture between a pH of 6.5 and 5.5. The production profile was similar within this pH range, and thus a pH of 6 was selected for subsequent incubations.

### Effect of organic acid concentrations

**Fig. 3:**
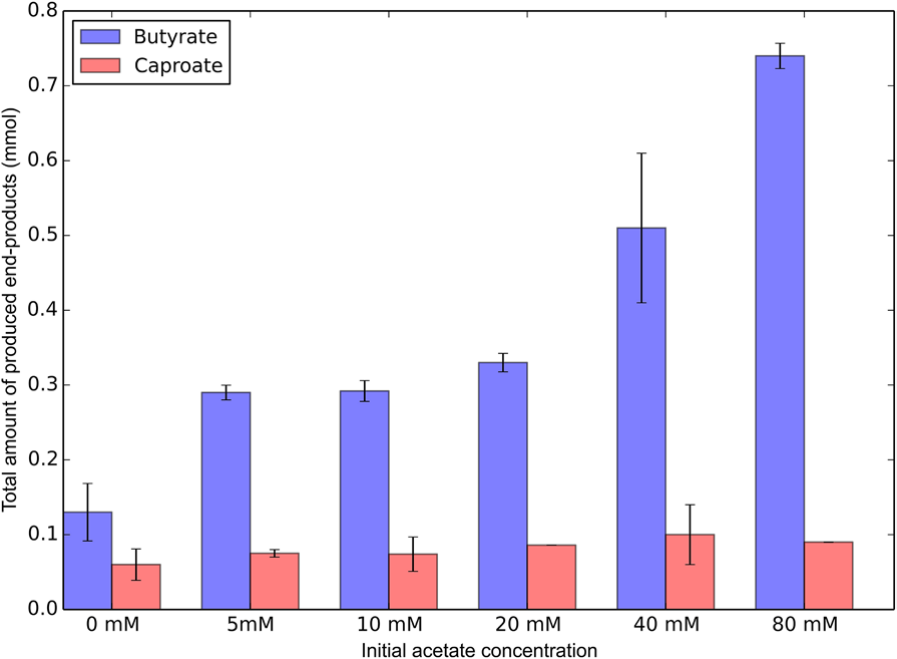
Effect of initial acetate concentration on the production of MCFAs. Data displayed are representative for 13 days after incubation using 130 kPa CO as a substrate. At the end of cultivation, CO was depleted in all cultures. On all *graphs*, a standard deviation is displayed over duplicate experiments

Increasing initial acetate concentration in the medium, from 0 to 80 mM, resulted in a significant increase in butyrate production (Fig. 3). Co-cultures incubated without initial addition of acetate did grow, but were significantly slower and showed a lower butyrate yield after consuming the full CO headspace. Caproate production was not affected by the initial acetate levels.

The effect of different initial butyrate concentrations was tested in a range of 0–45 mM, of which the highest concentration is triple the amount reached under the standard incubation conditions (Fig. 2). No toxicity effects on the co-culture were observed in this range. However, butanol production was observed with increasing butyrate concentrations, reaching levels up to 6 mM when 45 mM butyrate was initially present. Initial caproate concentrations ranging from 0 to 35 mM were tested. Increasing caproate concentrations resulted in a longer lag phase, suggesting toxicity effects. Co-cultures incubated with initial caproate concentrations above 12 mM did not grow after 16 days of incubation, whereas controls initiated growth within the first 4 days of incubation. In cultures with 12 mM caproate, hexanol reached concentrations of 2.5 mM at the end of cultivation. Additionally, monocultures of *C. autoethanogenum* incubated with CO in the presence of initial butyrate or caproate formed butanol or hexanol, respectively.

### Effect of gas composition

**Fig. 4:**
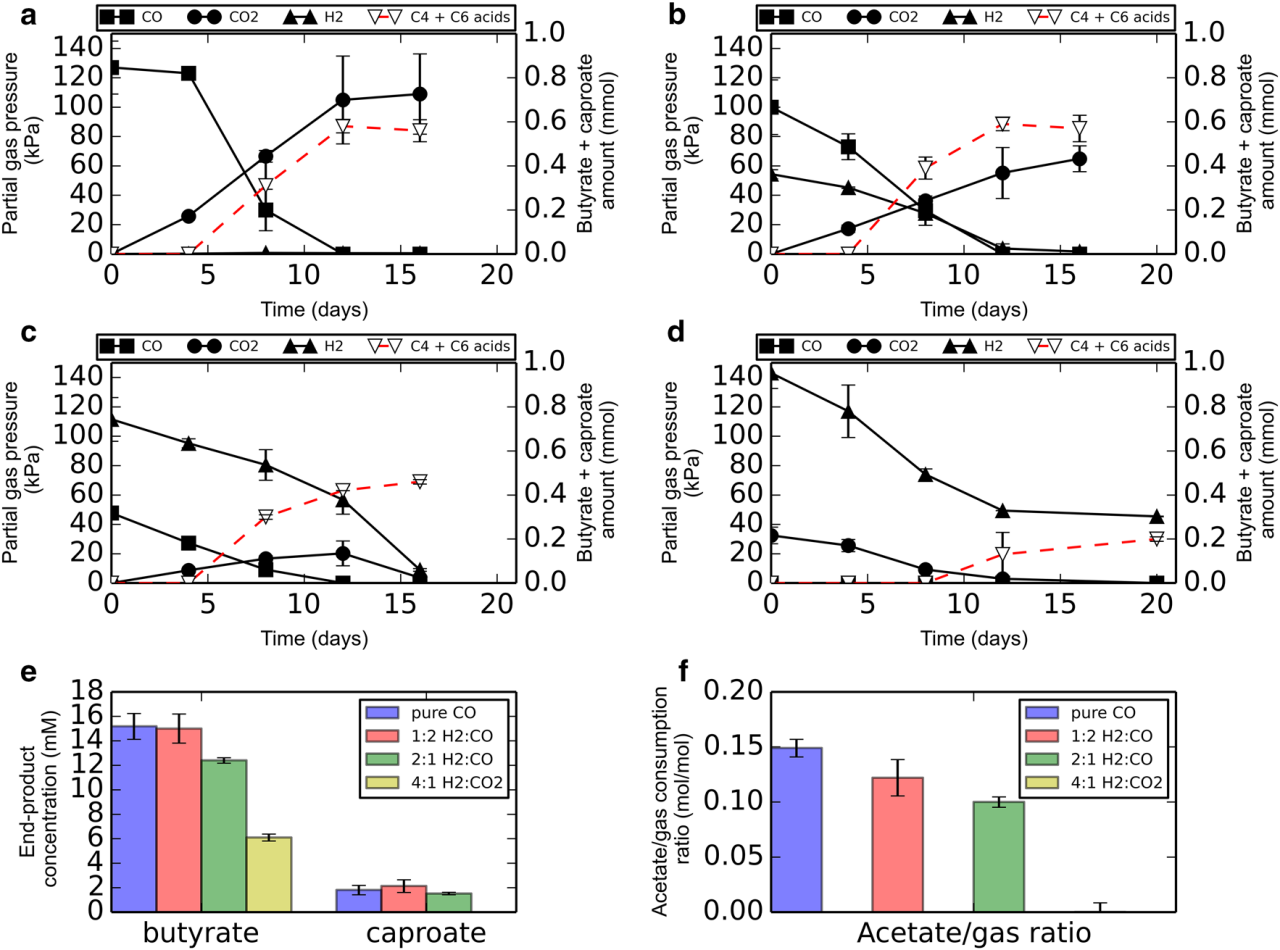
The effect of H_2_:CO ratio on the production profile of the co-culture. **a** Pure CO headspace. **b** 1:2 ratio of H_2_/CO **c** 2:1 ratio of H_2_:CO. **d** H_2_/CO_2_ headspace. **e** Product concentrations at the end of incubation. **f** Mole of acetate consumed per mole of gas (H_2_ + CO) consumed. On all *graphs* a standard deviation is displayed over duplicate experiments. *Solid* and *open circle* symbols represent *left* and *right y-axis*, respectively

To assess if syngas could be a potential donor for the co-culture, the effect of different H_2_/CO ratios was tested under non-shaking conditions (Fig. 4). Hydrogen and CO were co-utilized and resulted in similar end products as from CO alone. Incubations with 80:20 H_2_/CO_2_ sustained the co-culture (Fig. 4d), producing butyrate, but no caproate. Additionally, production rates and end-concentrations were lower when compared with incubations with H_2_/CO. Co-cultures under a H_2_/CO_2_ headspace utilized both gasses, and after CO_2_ depletion consumption of H_2_ stopped. Cultures with a higher CO/H_2_ ratio produced relatively more chain-elongated products, compared to cultures containing relatively less CO (Fig. 4e). Additionally, cultures with higher CO/H_2_ ratio utilized more acetate per mole of gas consumed (Fig. 4f).

### Enhancing productivity of the co-culture

**Fig. 5:**
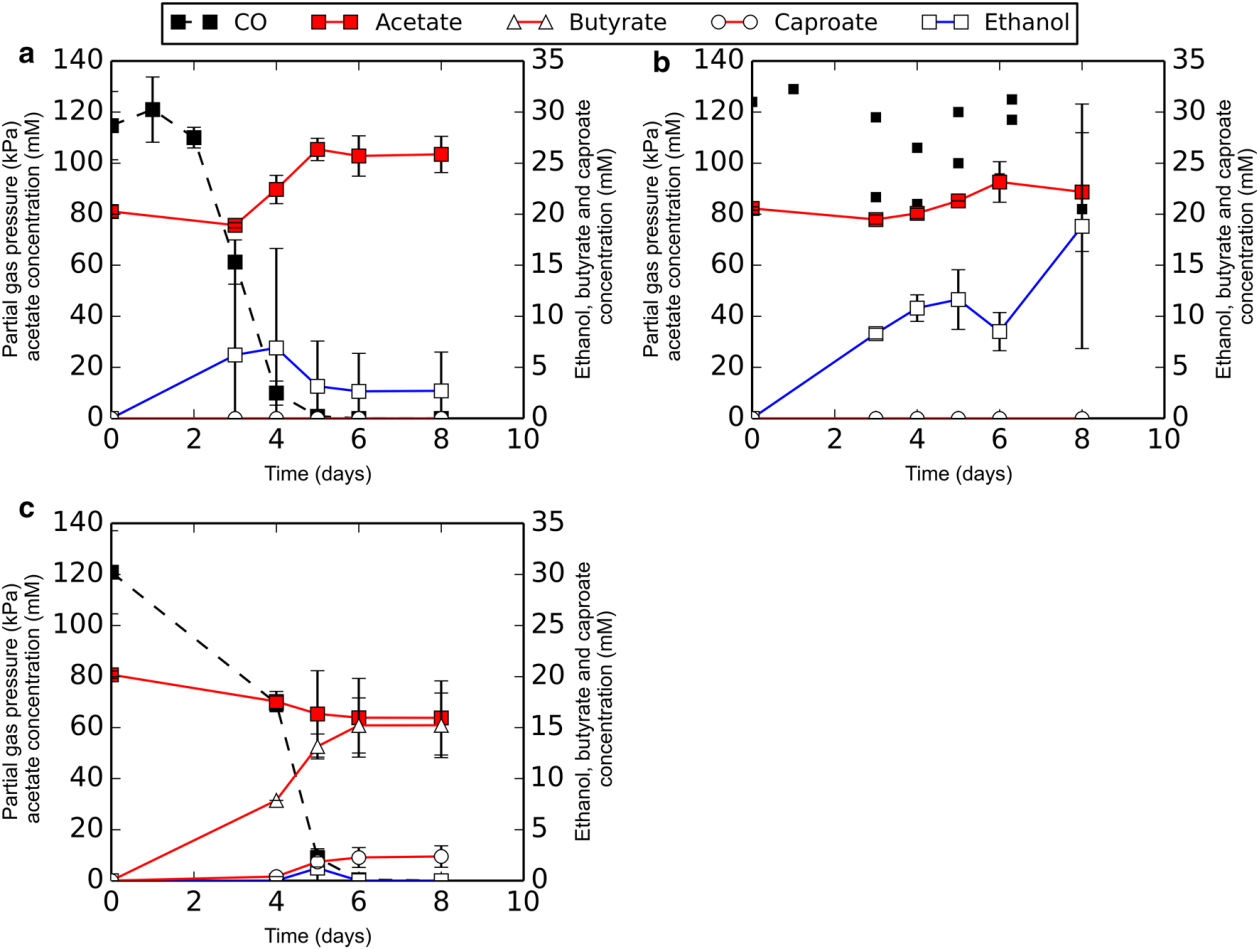
Effect of shaking and CO pressure on the co-culture. **a** Production profile under shaking conditions. **b** Production profile with maintained CO pressure (>50 kPa), under shaking conditions. **c** Production profile after initial non-shaking incubation and subsequent transfer to shaking conditions (after *t* = 4). On all data series, a standard deviation is displayed over duplicate experiments. *Solid* and *open circle* symbols represent *left* and *right y-axis*, respectively

Co-cultures put under shaking conditions initially produced ethanol and acetate, but did not show butyrate and caproate formation (Fig. 5a). Instead, these incubations converted ethanol back to acetate upon reaching low CO pressures in the headspace. Cultivation with CO pressure maintained above 50 kPa during shaking cultivation resulted in less oxidation of ethanol back to acetate (Fig. 5b). The fact that no MCFA were produced indicates that *C. kluyveri* activity is inhibited. Re-oxidation of ethanol to acetate at the end of the experiment is likely performed by the metabolically active *C. autoethanogenum*. Initiating co-cultivation under non-shaking conditions, followed by transfer to shaking conditions after butyrate production was detected, which resulted in a functional co-culture (Fig. 5c).

**Fig. 6:**
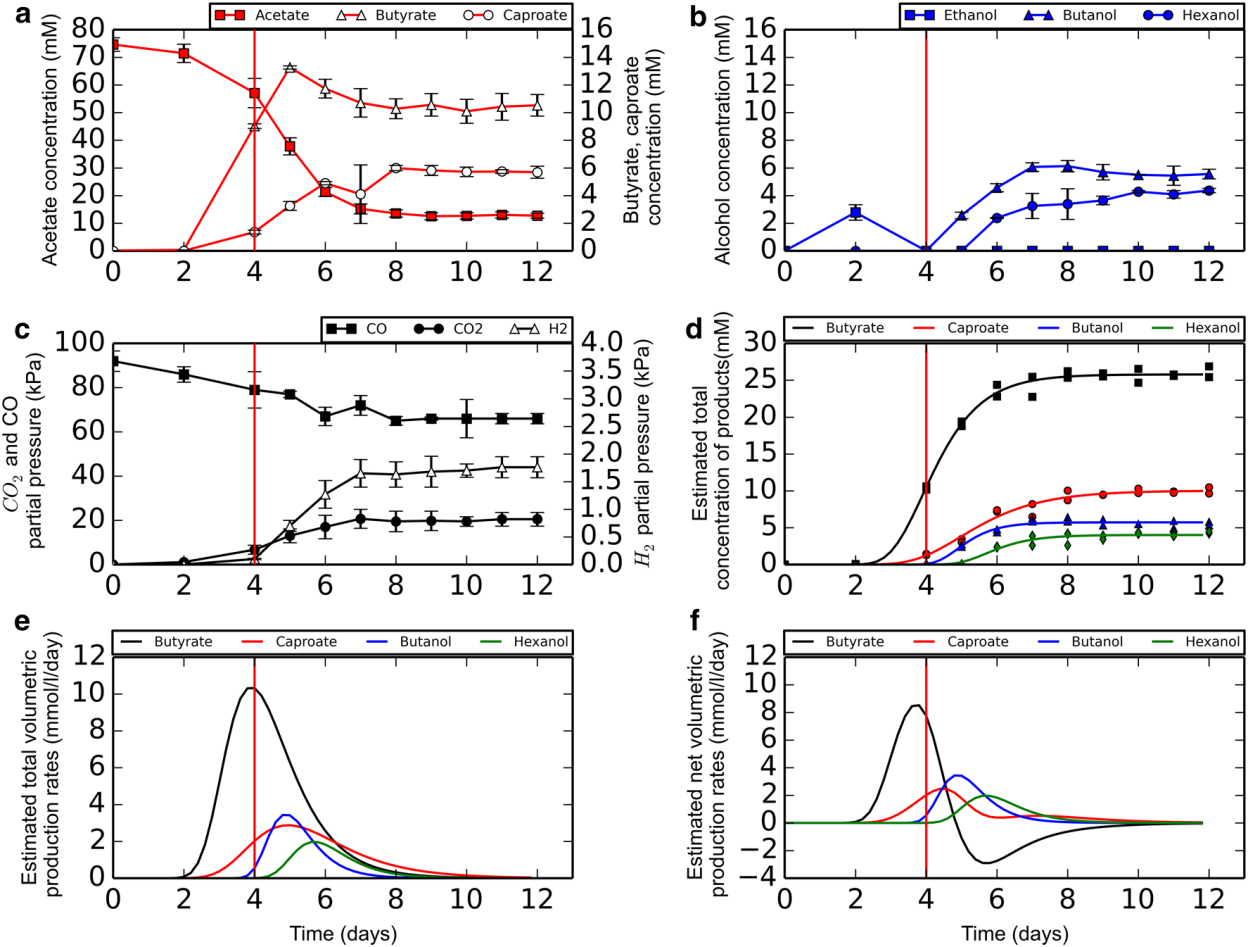
Co-cultivation under excess CO conditions. Shaking was applied after 4 days (*red vertical line*). **a** Acid concentration profile. **b** Alcohol concentration profile. **c** Partial gas pressures of CO, CO_2_, and H_2_. **d** Estimated total concentration of products formed, approximated by a Gompertz equation. **e** Total estimated volumetric production rates displayed as the derivative of the Gompertz equation. **f** Estimated net volumetric production rates after compensation of product formation and consumption according to Eqs. 3 and 4. *Solid* and *open circle* symbols represent *left* and *right y-axis*, respectively

Production potential of the co-culture under CO-excess and shaking conditions was tested. During the non-shaking phase of incubation, mainly butyrate and caproate were produced (Fig. 6a–c). Upon applying shaking conditions, production of these products further increased and additional production of butanol and hexanol was observed. MCFA or alcohols with a length longer than C_6_ were not detected. After 2 days of shaking, the pH of the culture had increased from 6.0 to 7.2. After this point, CO-consumption rates declined and production rates dropped. Eventually, production stopped before CO had been depleted. In order to assess the production rates of the co-culture, the data were fitted to a modified Gompertz equation [19]. As butyrate can act as acceptor molecule in caproate formation and both acids are precursors for their respective alcohols, their total production is masked by the production of other compounds as displayed by Eqs. 3 and 4. The estimated total product concentrations were fitted to the model (Fig. 6d; Table 2). The derivative of the obtained Gompertz equation (Eq. 2) was used to estimate the total volumetric production rates of each of the compounds in time (Fig. 6e). The net volumetric production rate was approximated by compensating the total volumetric production rate for the volumetric production rate of subsequent products (Fig. 6f).

**Table 2:** Gompertz model (Eqs. 1, 2) parameter estimates, and their standard errors, for each of the products

	Butyrate	Caproate	Butanol	Hexanol
A (mM)^a^	25.8 (±0.24)	10.0 (±0.25)	5.73 (±0.12)	4.01 (±0.16)
*V*_m_ (mmol/l/day)^b^	10.4 (±0.80)	2.86 (±0.31)	3.47 (±0.69)	1.98 (±0.46)
*γ* (days)^c^	2.99 (±0.11)	3.69 (±0.19)	4.28 (±0.18)	4.95 (±0.26)

^a^ Maximal product concentration

^b^ Maximal volumetric production rate

^c^ Lag time before production occurs

3 $$\left[ {\text{butyrate}} \right]_{{_{\text{total}} }} = \left[ {\text{butyrate}} \right]_{{_{{_{\text{observed}} }} }} + \left[ {\text{caproate}} \right]_{{_{{_{\text{observed}} }} }} + \left[ {\text{butanol}} \right]_{{_{{_{\text{observed}} }} }} + \left[ {\text{hexanol}} \right]_{{_{{_{\text{observed}} }} }}$$

4 $$\left[ {\text{caproate}} \right]_{{_{\text{total}} }} = \left[ {\text{caproate}} \right]_{{_{{_{\text{observed}} }} }} + \left[ {\text{hexanol}} \right]_{\text{observed}}$$

The maximal production rate for butyrate is approximately 8.5 ± SE 1.1 mmol/l/day. Caproate reaches a maximal net production rate of 2.5 ± SE 0.63 mmol/l/day. Butanol and hexanol are the last to be formed at maximal production rates of 3.5 ± SE 0.69 and 2.0 ± SE 0.46 mmol/l/day, respectively.

## Discussion

The co-culture of *C. autoethanogenum* and *C. kluyveri* is capable of converting CO or syngas to a mixture of C_4_ and C_6_ fatty acids and their respective alcohols. Monocultures of *C. kluyveri* are unable to utilize CO and its metabolism is even inhibited by it. Nonetheless, activity of *C. kluyveri* is observed in the co-culture in presence of 130 kPa CO. *C. autoethanogenum* likely facilitates growth of *C. kluyveri*, by removing CO from the liquid. This is analogous to the theorized role of thermophilic carboxydotrophs in volcanic environments, creating a niche for non-CO-tolerant organisms [22]. This additionally explains the inability of the co-culture to grow instantly in shaking conditions. Low biomass levels at the start combined with increased CO mass transfer under shaking conditions, cause inhibition of *C. kluyveri*, resulting in growth of *C. autoethanogenum* only (Fig. 5). Cultivation under non-shaking conditions allows both organisms to initiate growth, eventually allowing shaking conditions.

### Effect of environmental factors on co-culture functionality

Ethanol is the driving compound for chain elongation, making it a key intermediate in the co-culture. Its production is observed at the start of cultivation, but concentrations quickly decrease to levels below the detection limit when butyrate and caproate were formed (Figs. 2, 6b). This suggests that ethanol production is the limiting factor for chain elongation. Several environmental factors were expected to increase ethanol production of the carboxydotrophic strain. Two of these factors are lowering of pH and decreasing concentration of yeast extract [23]. However, we observed no clear differences in production within the tested range of viable pH and yeast extract concentrations of the co-culture.

Ethanol production in acetogenic carboxydotrophs can occur directly via acetyl-CoA or indirectly via acetate [7, 24]. When *C. ljungdahlii* is grown on CO, it expresses an aldehyde oxidoreductase (AOR), required for the indirect ethanol production pathway. Upon addition of external acids, AORs were found more abundantly expressed, indicating upregulation of the indirect alcohol production pathways [25]. In the co-culture, we observed increased butyrate production upon addition of acetate (Fig. 3), which indirectly indicates that ethanol production is stimulated. Similarly, the presence of butyrate or caproate stimulated the production of their respective alcohols. This suggests that, as observed in pure cultures of *C. ljungdahlii* [12, 25], alcohol production in this co-culture is stimulated by the presence of their respective acids. This could be a stress response to the presence of relatively more acids in the undissociated form, which can be considered toxic, stimulating conversion to their respective alcohols. Additionally, the acids could act as an electron sink, to counter the strong reducing pressure of CO, which would explain the formation of relatively more alcohols in the shaking cultures (Fig. 6b). Presence of high concentrations of acetate thus serves a double purpose: (i) substrate for chain elongation and (ii) stimulation of the formation of ethanol. The co-culture was functional in absence of acetate, indicating that the synthetic community can sustain itself on purely CO as a substrate. However, production rates under these conditions were significantly lower.

When incubating instantly under shaking conditions, the oxidation of ethanol to acetate was observed when CO became depleted (Fig. 5a). As no chain elongation activity is observed, *C. autoethanogenum* appears responsible for the ethanol oxidizing activity, potentially utilizing it as an alternative electron donor to produce acetate. Such a metabolism was observed for the acetogen *Acetobacterium woodii,* utilizing ethanol for production of acetate [26]. Oxidation of ethanol to acetate by *C. autoethanogenum* was partly suppressed under maintained CO pressure (Fig. 5b). The maintained CO pressure potentially causes the cells to be more reduced, forcing the reaction toward production of ethanol to maintain proper redox balance.

When applying syngas technology, all the gas is preferably converted to soluble products, leaving no CO_2_ in the exhaust gas. Presence of hydrogen in the headspace allows for additional fixation of CO_2_, which makes hydrogen an interesting component to completely remove CO without CO_2_ exhaust. Under the tested conditions, a 2:1 ratio of H_2_:CO appears to be close to optimal as almost all the gaseous compounds are converted to soluble products after depletion of electron donor, releasing no net CO_2_ (Fig. 4c). However, the amount of chain-elongated products formed is slightly decreased under these conditions as larger amounts of H_2_ appear to be required to obtain similar results as with CO (Fig. 4e). This might be related to the lower redox potential of CO (*E*^0′^ = −520 mV) compared to hydrogen (*E*^0′^ = −414 mV). This allows for the production of relatively more reduced products such as ethanol [27–29], which can subsequently be used as a substrate for chain elongation. At lower CO/H_2_ ratios, formation of ethanol is expected to be less favorable, resulting in relatively more acetate formation. This is supported by the lowered net amount of acetate consumed per mole of gas used (Fig. 4f).

### Co-culture limitations

In cultures incubated under shaking conditions with excess CO, more acetate is consumed than is required for chain elongation (Fig. 6; Table 1). This suggests that acetate is mainly converted to ethanol. This is supported by the observation that a major part of the oxidized CO can be found back as CO_2_ (Fig. 6c), indicating that CO mainly acts as an electron donor for the reduction of acetate to ethanol (Table 1). This rapid conversion of acetate to ethanol and the production of higher alcohols from the generated medium-chain fatty acids (Fig. 6b), likely caused the observed pH increase from 6.0 to 7.2. *C. autoethanogenum* was reported to grow in a pH range of 4.5–6.5 with an optimum of 6 [15]. *C. kluyveri* was reported to grow within a pH range of 6.0–7.5 with an optimum of 6.8 [30]. The pH reached during co-cultivation exceeds 6.5 and thus likely causes inhibition of *C. autoethanogenum*. Resulting in a decrease in activity of the co-culture before CO was depleted. Additionally, the pH of the medium also affects the amount of chain-elongated products that can be accumulated. Caproate toxicity is a general problem in chain elongation processes and is strongly pH dependent, as the toxicity effect is caused by the undissociated form [31]. A mixed culture bioreactor, fed with ethanol effluent from a syngas reactor, tolerated 3 mM caproate at pH 5.4. The undissociated fraction at this pH is 22 %, which equals 0.66 mM [32]. Reactors operated at a higher pH or reactors with continuous removal of caproate allowed a higher accumulation and higher production rates, respectively [33, 34]. *C. kluyveri* strain 3231B was found to accumulate caproate to levels of 110 mM at pH 6.8 [16], which translates into an undissociated fraction of 1.3 mM. The co-culture of *C. kluyveri* and *C. autoethanogenum*, at pH 6, tolerated 12 mM caproate. Under these conditions 7 % is in undissociated form (approx. 0.88 mM), which is in the same order of magnitude of the numbers reported for other cultures.

Growth performance of the individual strains and chain-elongated product toxicity are thus both strongly affected by pH. More acidic environments stimulate the growth of *C. autoethanogenum*, but inhibit *C. kluyveri* and promote toxicity of caproate. A higher pH allows for higher caproate concentrations but inhibits *C. autoethanogenum*. Therefore, controlling pH between 5.5 and 6.5 appears essential for maintaining a well-performing co-culture.

### Co-culture assessment and comparison

**Fig. 7:**
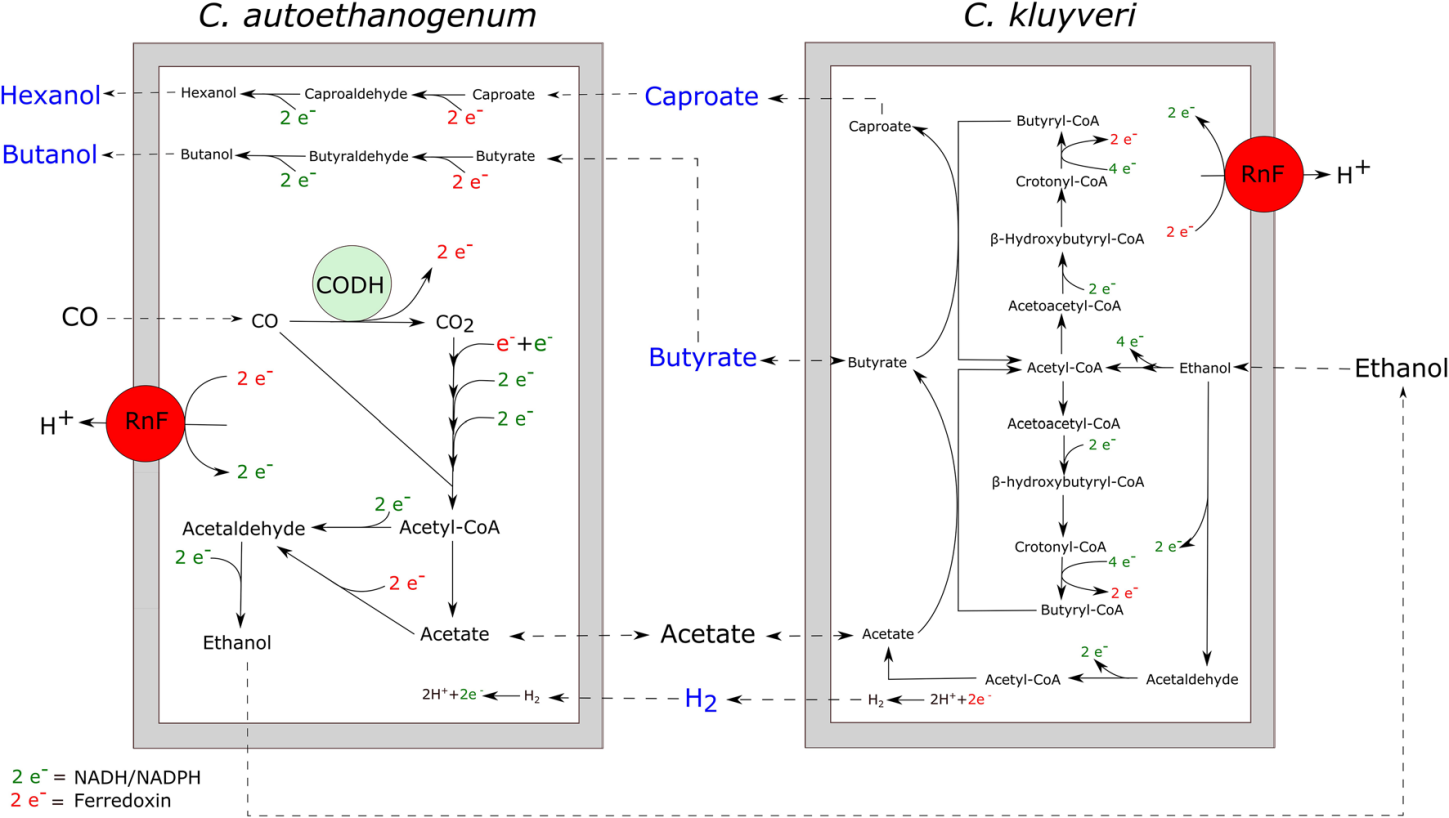
Schematic representation of the co-culture of *C. autoethanogenum* and *C. kluyveri*. Reaction stoichiometry and ATP yield for each of the cells are not displayed. Conversion of butyrate to butyraldehyde and caproate to caproaldehyde is assumed to proceed via an aldehyde oxidoreductase, as is observed for acetate to acetaldehyde formation. *CODH* carbon monoxide dehydrogenase, *RnF* ferredoxin-NAD:oxidoreductase

Based on the pure culture incubations, *C. autoethanogenum* produces ethanol and acetate from CO. *C. kluyveri* is not able to utilize CO. Butyrate and caproate are not observed to be generated by *C. autoethanogenum* in pure culture containing CO, ethanol, acetate, or a combination of the substrates. Production of these MCFAs can thus solely be assigned to *C. kluyveri*. Pure culture incubation of *C. autoethanogenum* with CO and butyrate or caproate resulted in butanol and hexanol production. Production of these alcohols was never observed in any of the tested pure cultures of *C. kluyveri*. Hydrogen can be formed by both members of the co-culture, but appears to be only utilized by *C. autoethanogenum*. Taking these factors into account, a model system with solely CO as an input, generating butyrate, caproate, butanol, and hexanol as the end products can be proposed (Fig. 7).

Only few reports describe microbial systems producing MCFAs and/or higher alcohols from carbon monoxide or syngas. Mixed cultures in a H_2_/CO_2_-fed membrane bioreactor, dominated by *Clostridium* species, produced fatty acids up to C_8_ length [18]. However, this system lacked CO in the inflow-gas, which is a major component in non-pretreated syngas. The lower redox pressure of H_2_/CO_2_ compared to CO-containing syngas might be a main reason for the relatively low production rates and absence of longer chain alcohols reported in the previous system (Table 3). *Clostridium carboxidivorans* is one of the organisms known to be capable of producing chain-elongated acids and their respective alcohols from CO in pure culture (Table 3). Production rates of the alcohols by *C. carboxidivorans* appear to increase at sub-optimal growth temperatures [13]. The co-culture described here, currently has production rates comparable to the pure cultures of *C. carboxidivorans* (Table 3). However, the functioning of the co-culture is not fully explored and several parameters can still be optimized, such as pH control, medium composition, and gas composition/mass transfer. Therefore, we expect the production potential of the co-culture can be increased, potentially becoming interesting for syngas-based applications.

**Table 3:** Maximal (M) and average (A) production rates (mmol/l/day) of the co-culture compared with other pure- and mixed cultures

	Acetate	Butyrate	Caproate	Ethanol	Butanol	Hexanol	Substrate	Reference
Co-culture (M)^a^	NA	8.5	2.5	NA	3.5	2.0	Acetate + CO	This study
Co-culture (A)^a, b^	NA	4.2	0.7	NA	1.4	0.9	Acetate + CO	This study
*Clostridium carboxidivorans*(A)^a, c^	0.8	0.25	0.2	3.0	1.0	0.6	CO + H_2_	[6]
*Clostridium carboxidivorans* P7 (M)^a, d^	47	6.3	4.6	8.1	2.7	2.1	CO + H_2_	[13]
*Clostridium carboxidivorans* P7 (A)^a, d^	2.3	0.89	0.48	1.57	0.53	0.25	CO + H_2_	[13]
*Clostridium carboxidivorans* (M)^a, e^	NA	NA	ND	16.7	4.5	ND	CO	[11]
Mixed culture on H_2_/CO_2_^a, f^	3.25	0.65	0.26	ND	ND	ND	H_2_ + CO_2_	[18]

^a^ Zero or negative rates are indicated NA, not determined rates are indicated ND

^b^ Average production rates in this study were calculated over the production stage with net rates above 0.05 mmol/l/day, in this way the lag phase and inhibited phase, in which there is no significant production, are neglected

^c^ Rates were recalculated from given concentrations assuming a production phase of 360 h. The data displayed represent the culture labeled as −Cu/+10 × Mo

^d^ Rates reported were originally in mmol/g protein/h and were here recalculated to the maximal and average volumetric production rates using the maximal and average value for the protein concentration and specific production rate reported, respectively. Data shown are taken from the culture growing at 25 °C in exponential phase

^e^ Rates were recalculated from given specific production rates in (g/g biomass/h). A value 0.2 g/l biomass was used for performing the recalculation

^f^ Rates were recalculated from given volumetric production rates in (mmol-C/l/day). Data displayed here represent the maximal reported production rates in different phases of the cultivation

## Conclusion

The synthetic co-culture of *C. autoethanogenum* and *C. kluyveri* is able to convert carbon monoxide and syngas to a mixture of medium-chain fatty acids and their respective alcohols. Despite the toxic effect of CO on *C. kluyveri*, chain elongation activity was found in the co-culture, indicating that CO toxicity is relieved by the presence of a carboxydotrophic organism. The culture grows without addition of ethanol and acetate, but the presence of acetate significantly stimulated production. The co-culture was limited by the quickly increasing pH as a result of solventogenic reactions. Additionally, caproate concentration can be an inhibitory factor, of which the toxicity effect is a trade-off between pH and concentration. Overall this co-culture is a proof-of-principle that the carboxylate and syngas platform can be integrated in one growth vessel, and could become a promising way to enhance the production of MCFAs and their respective alcohols from syngas.

## Abbreviations

AOR: aldehyde oxidoreductase
CODH: carbon monoxide dehydrogenase
CO: carbon monoxide
CO_2_: carbon dioxide
H_2_: hydrogen
MCFA: medium-chain fatty acid
Syngas: synthesis gas

## References

[1] Dürre P, Eikmanns BJ (2015). C1-carbon sources for chemical and fuel production by microbial gas fermentation. Curr Opin Biotechnol.

[2] Frost LJ, Hartvigsen J, Elangovan S (2010). Formation of synthesis gas using solar concentrator photovoltaics (SCPV) and high temperature co-electrolysis (HTCE) of CO_2_ and H_2_O. Offshore Technol Conf..

[3] Latif H, Zeidan AA, Nielsen AT, Zengler K (2014). Trash to treasure: production of biofuels and commodity chemicals via syngas fermenting microorganisms. Curr Opin Biotechnol.

[4] Jeong J, Bertsch J, Hess V, Choi S, Choi IG, Chang IS, Müller V (2015). A model for energy conservation based on genomic and experimental analyses in a carbon monoxide-utilizing, butyrate-forming acetogen, *Eubacterium limosum* KIST612. Appl Environ Microbiol.

[5] Worden R, Grethlein A, Zeikus J, Datta R (1989). Butyrate production from carbon monoxide by *Butyribacterium methylotrophicum*. Appl Biochem Biotechnol.

[6] Phillips JR, Atiyeh HK, Tanner RS, Torres JR, Saxena J, Wilkins MR, Huhnke RL (2015). Butanol and hexanol production in *Clostridium carboxidivorans* syngas fermentation: medium development and culture techniques. Biores Technol.

[7] Köpke M, Held C, Hujer S, Liesegang H, Wiezer A, Wollherr A, Ehrenreich A, Liebl W, Gottschalk G, Durre P (2010). *Clostridium ljungdahlii* represents a microbial production platform based on syngas. PNAS.

[8] Berzin V, Tyurin M, Kiriukhin M (2013). Selective n-butanol production by Clostridium sp. MTButOH1365 during continuous synthesis gas fermentation due to expression of synthetic thiolase, 3-hydroxy butyryl-CoA dehydrogenase, crotonase, butyryl-CoA dehydrogenase, butyraldehyde dehydrogenase, and NAD-dependent butanol dehydrogenase. Appl Biochem Biotechnol.

[9] Liu K, Atiyeh HK, Stevenson BS, Tanner RS, Wilkins MR, Huhnke RL (2014). Continuous syngas fermentation for the production of ethanol, n-propanol and n-butanol. Biores Technol.

[10] Liu K, Atiyeh HK, Stevenson BS, Tanner RS, Wilkins MR, Huhnke RL (2014). Mixed culture syngas fermentation and conversion of carboxylic acids into alcohols. Biores Technol.

[11] Fernández-Naveira Á, Abubackar HN, Veiga MC, Kennes C (2016). Efficient butanol-ethanol (BE) production from carbon monoxide fermentation by *Clostridium carboxidivorans*. Appl Environ Microbiol.

[12] Perez JM, Richter H, Loftus SE, Angenent LT (2013). Biocatalytic reduction of short-chain carboxylic acids into their corresponding alcohols with syngas fermentation. Biotechnol Bioeng.

[13] Ramió-Pujol S, Ganigué R, Bañeras L, Colprim J (2015). Incubation at 25° C prevents acid crash and enhances alcohol production in *Clostridium carboxidivorans* P7. Biores Technol.

[14] Cho C, Jang YS, Moon HG, Lee J, Lee SY (2015). Metabolic engineering of clostridia for the production of chemicals. Biofuels Bioprod Bioref.

[15] Abrini J, Naveau H, Nyns E-J (1994). *Clostridium autoethanogenum*, sp. nov., an anaerobic bacterium that produces ethanol from carbon monoxide. Arch Microbiol.

[16] Weimer PJ, Stevenson DM (2012). Isolation, characterization, and quantification of *Clostridium kluyveri* from the bovine rumen. Appl Environ Microbiol.

[17] Weimer PJ, Nerdahl M, Brandl DJ (2015). Production of medium-chain volatile fatty acids by mixed ruminal microorganisms is enhanced by ethanol in co-culture with *Clostridium kluyveri*. Biores Technol.

[18] Zhang F, Ding J, Zhang Y, Chen M, Ding ZW, van Loosdrecht MC, Zeng RJ (2013). Fatty acids production from hydrogen and carbon dioxide by mixed culture in the membrane biofilm reactor. Water Res.

[19] Zwietering M, Jongenburger I, Rombouts F, Van’t Riet K (1990). Modeling of the bacterial growth curve. Appl Environ Microbiol.

[20] Bornstein B, Barker H (1948). The nutrition of *Clostridium kluyveri*. J Bacteriol.

[21] Martin ME, Richter H, Saha S, Angenent LT (2015). Traits of selected Clostridium strains for syngas fermentation to ethanol. Biotechnol Bioeng.

[22] Techtmann SM, Colman AS, Robb FT (2009). ‘That which does not kill us only makes us stronger’: the role of carbon monoxide in thermophilic microbial consortia. Environ Microbiol.

[23] Abubackar HN, Veiga MC, Kennes C (2012). Biological conversion of carbon monoxide to ethanol: effect of pH, gas pressure, reducing agent and yeast extract. Biores Technol.

[24] Köpke M, Mihalcea C, Bromley JC, Simpson SD (2011). Fermentative production of ethanol from carbon monoxide. Curr Opin Biotechnol.

[25] Xie B-T, Liu Z-Y, Tian L, Li F-L, Chen X-H (2015). Physiological response of *Clostridium ljungdahlii* DSM 13528 of ethanol production under different fermentation conditions. Biores Technol.

[26] Bertsch J, Siemund AL, Kremp F, Müller V (2015). A novel route for ethanol oxidation in the acetogenic bacterium *Acetobacterium woodii*: the AdhE pathway. Environ Microbiol.

[27] Bertsch J, Müller V (2015). Bioenergetic constraints for conversion of syngas to biofuels in acetogenic bacteria. Biotechnol Biofuels.

[28] Diender M, Stams AJ, Sousa DZ (2015). Pathways and bioenergetics of anaerobic carbon monoxide fermentation. Front Microbiol.

[29] Hurst KM, Lewis RS (2010). Carbon monoxide partial pressure effects on the metabolic process of syngas fermentation. Biochem Eng J.

[30] Barker H, Taha S (1942). *Clostridium kluyverii*, an organism concerned in the formation of caproic acid from ethyl alcohol. J Bacteriol.

[31] Steinbusch KJJ, Hamelers HVM, Plugge CM, Buisman CJN (2011). Biological formation of caproate and caprylate from acetate: fuel and chemical production from low grade biomass. Energy Environ Sci.

[32] Vasudevan D, Richter H, Angenent LT (2014). Upgrading dilute ethanol from syngas fermentation to n-caproate with reactor microbiomes. Biores Technol.

[33] Agler MT, Wrenn BA, Zinder SH, Angenent LT (2011). Waste to bioproduct conversion with undefined mixed cultures: the carboxylate platform. Trends Biotechnol.

[34] Grootscholten TI, Steinbusch KJ, Hamelers HV, Buisman CJ (2013). Chain elongation of acetate and ethanol in an up flow anaerobic filter for high rate MCFA production. Biores Technol.

